# Relation between Arterial Stiffness and Markers of Inflammation and Hemostasis – Data from the Population-based Gutenberg Health Study

**DOI:** 10.1038/s41598-017-06175-2

**Published:** 2017-07-24

**Authors:** Natalie Arnold, Tommaso Gori, Renate B. Schnabel, Andreas Schulz, Jürgen H. Prochaska, Tanja Zeller, Harald Binder, Norbert Pfeiffer, Manfred Beutel, Christine Espinola-Klein, Karl J. Lackner, Stefan Blankenberg, Thomas Münzel, Philipp S. Wild

**Affiliations:** 1 Center for Cardiology I, Center for Cardiology, University Medical Center of the Johannes Gutenberg-University Mainz, Mainz, Germany; 2 Preventive Cardiology and Preventive Medicine, Center for Cardiology, University Medical Center of the Johannes Gutenberg-University Mainz, Mainz, Germany; 3 Center for Translational Vascular Biology (CTVB), University Medical Center of the Johannes Gutenberg-University Mainz, Mainz, Germany; 4 DZHK (German Center for Cardiovascular Research), Partner Site RhineMain, Mainz, Germany; 5 Department of General and Interventional Cardiology, University Medical Center Hamburg-Eppendorf, Hamburg, Germany; 6 DZHK (German Center for Cardiovascular Research), Partner Site Hamburg/Kiel/Lübeck, Hamburg, Germany; 7 Center for Thrombosis and Hemostasis, University Medical Center of the Johannes-Gutenberg University Mainz, Mainz, Germany; 8 Institute of Medical Biostatistics, Epidemiology and Informatics (IMBEI), University Medical Center of the Johannes Gutenberg-University Mainz, Mainz, Germany; 9 Department of Ophthalmology, University Medical Center of the Johannes Gutenberg-University Mainz, Mainz, Germany; 10 Department of Psychosomatic Medicine and Psychotherapy, University Medical Center of the Johannes Gutenberg-University Mainz, Mainz, Germany; 11 Institute for Clinical Chemistry and Laboratory Medicine of the Johannes Gutenberg-University Mainz, Mainz, Germany

## Abstract

The relation between inflammation, hemostasis and arterial stiffness is of pathophysiological relevance for the development of cardiovascular disease (CVD). Data investigating this interplay using stiffness index (SI) by digital photoplethysmography are not available yet. Therefore, sex-specific relation between SI and inflammatory and hemostatic biomarkers was investigated within 13,724 subjects from the population-based Gutenberg Health Study. C-reactive protein (CRP), white blood cell count (WBCC), neopterin, interleukin-18, interleukin-1 receptor antagonist (IL-1RA), fibrinogen and hematocrit were measured. Multivariable linear regression analysis with adjustment for cardiovascular risk factors, medication, and hormonal status (in females) revealed an independent association between SI and WBCC, IL-1RA and hematocrit in both sexes, and with fibrinogen in women. There was a joint effect of increasing tertiles of SI and biomarker concentrations for future CVD risk prediction. Subjects with both SI and biomarker concentration above the median had the worst overall survival and with both below the median the best survival during a follow-up period of 6.2 ± 1.7 years, except for hematocrit. The results support the relation between inflammation, hemostasis and arterial stiffness measured by digital photoplethysmography. Markers of inflammation and hemostasis modulate the ability of SI to identify subjects at risk for future CVD or higher mortality.

## Introduction

High blood pressure represents one of the potent cardiovascular risk (CV) factor and a leading cause of total and CV mortality^1, 2^. Recently published studies implicated a role of inflammation in the development of hypertension^3^. It has been further suggested that inflammation might be related to the stiffening of arteries, a condition associated with a normal ageing process or with premature vascular ageing, which occurs in hypertension and numerous metabolic disorders. Possible mechanisms between inflammation and arterial stiffness (AS) might be related to changes within the vessel wall such as e.g. inflammatory cell infiltration or vascular dysfunction^4^. Furthermore, various pro-inflammatory mediators and markers of oxidative stress promote an increased production of matrix metalloproteinases with subsequent degeneration of compliant elastin fibers that, in turn lead to decreased arterial compliance^5, 6^. Inflammation might also induce changes in the vascular smooth muscle phenotype with enhanced expression of osteoblasts, resulting in media calcification and subsequently stiffer arteries^7^.

To date several studies investigated the relation of AS with markers of inflammation and hemostasis in apparently healthy individuals with controversial results^8–17^. Most of published data were based on the measurement of carotid-femoral pulse wave velocity (cf-PWV), a widely used method for determination of AS to date^18^. Another interesting approach for assessment of arterial function and AS in particular might represent stiffness index (SI) by digital photoplethysmography^19^, which might reflect systemic AS^20, 21^, rather than providing similar to central PWV information. It represents easily performable and operator independent technique, which might be used in setting, where reliable simplicity of measurement is of relevance. So far, no study exists investigating a possible relationship between SI with markers of inflammation or hemostasis, neither in the general population nor among selected patient groups. Taking into account that SI was found to be markedly lower in females compared to males in our previous investigation^22^, we investigated such association sex-specifically in a large population-based Gutenberg Health Study (GHS).

## Results

**Figure 1:**
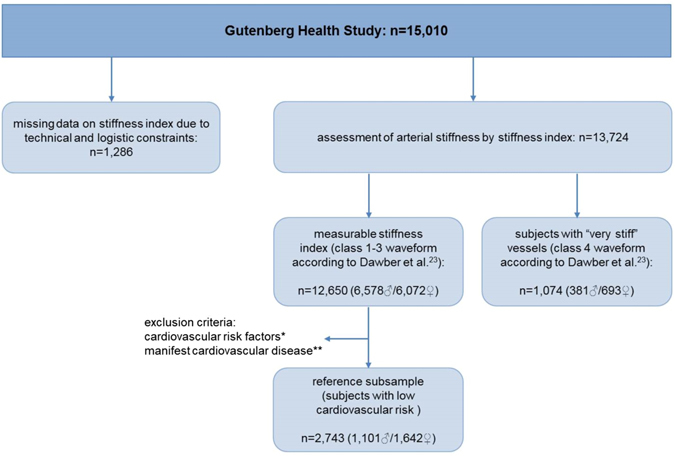
Study Design. *Cardiovascular risk factors: hypertension, obesity, dyslipidemia, smoking, diabetes, family history of myocardial infarction or stroke. **Manifest cardiovascular disease: history of myocardial infarction, coronary artery disease, congestive heart failure, stroke or peripheral artery disease.

From the overall population sample (n = 15,010), 1,286 individuals had missing values for SI due to logistical or technical constraints; the characteristics of these persons did not differ significantly from the sample investigated (data not shown). Therefore, assessment of AS was available in 13,724 subjects (Fig. 1). Among them, 1,074 individuals had no distinct notch in the waveform (so-called class 4 waveform according to Dawber *et al*.^23^.) and consequently no calculable SI values. In those subjects, AS was rated as “very stiff”. Within the remaining 12,650 subjects, SI was determined. The clinical characteristics of eligible subjects are summarized in Table 1.

**Table 1:** Demographic, Clinical and Laboratory Characteristics of the Population-based Sample (n = 13,724).

	Sample with assessment of arterial stiffness by digital photoplethysmography (n = 13,724)			
	Individuals with measurable stiffness index (n = 12,650)		Individuals with very stiff vessels* (n = 1,074)	
	Men	Women	Men	Women
n	6,578	6,072	381	693
Age, years	54.1 ± 11.0	54.5 ± 11.1	65.0 ± 7.4	62.9 ± 8.2
BMI, kg/m^2^	27.8 ± 4.2	26.7 ± 5.6	28.6 ± 4.5	27.7 ± 5.6
Systolic BP, mmHg	133 ± 16	128 ± 18	143 ± 20	139 ± 20
Diastolic BP, mmHg	84.0 ± 9.4	80.9 ± 9.3	82.1 ± 10.4	82.7 ± 10.2
Heart rate, bpm	67.9 ± 10.9	69.9 ± 10.2	68.8 ± 13.9	71.8 ± 12.4
Hypertension, %	52.7	41.3	79.0	72.1
Diabetes mellitus, %	8.7	5.1	21.1	9.7
Smoking, %	20.8	18.2	22.6	18.4
Dyslipidemia, %	36.5	21.1	43.4	31.3
Obesity, %	25.7	23.3	32.8	28.6
FH of MI/stroke, %	20.5	23.8	18.6	27.1
CAD^§^, %	5.8	1.8	16.8	4.9
MI^§^, %	4.0	1.2	13.1	3.3
CHF^§^, %	1.2	1.2	4.5	2.6
Stroke^§^, %	2.1	1.1	6.6	3.0
PAD^§^, %	3.1	3.0	14.2	4.9
CKD^§^ %	1.2	0.9	1.8	0.9
COPD^§^, %	4.2	5.6	6.8	5.3
OC intake, %	—	6.6	—	1.5
HRT, %	—	7.9	—	12.1
Menopause, %	—	64.6	—	91.5
ESC SCORE^†^, %	2.0 (1.0/6.0)	1.0 (0/2.00)	6.5 (4.0/10.0)	3.0 (1.0/4.0)
FRS^‡^, %	15.6 (8.0/26.5)	6.2 (2.96/11.83)	32.8 (23.7/44.6)	13.5 (8.6/19.8)
SI, m/s	8.35 ± 2.32	6.66 ± 1.69	“very stiff”	“very stiff”
CRP, mg/l	1.4 (0.5/2.8)	1.6 (0.6/3.4)	2.4 (1.2/4.7)	2.1 (1.1/4.2)
WBCC, 10^9^/l	6.8 (5.7/8.2)	6.9 (5.9/8.3)	7.4 (6.3/9.0)	7.2 (6.0/8.6)
Neopterin, pmol/l^&^	5.5 (4.7/6.4)	5.3 (4.7/6.3)	5.9 (4.9/7.1)	5.6 (4.8/6.7)
IL-18, pg/ml^#^	243 (193/314)	206 (167/260)	257 (201/349)	213 (175/261)
IL-1RA, pg/ml^**^	309 (233/476)	330 (246/442)	332 (256/455)	355 (261/469)
Fibrinogen, mg/dl	315 (272/367)	325 (282/378)	355 (305/428)	350 (306/412)
Hematocrit, %	43.7 ± 2.9	40.2 ± 2.7	43.4 ± 3.5	40.8 ± 3.0

*No distinct notch in digital volume pulse waveform. ^§^Information self-reported and medical records. ^†^German version of the ESC SCORE. ^‡^Framingham general CVD Risk Score. ^&^n = 3,548 (1,886 men/1,662 women); ^#^n = 4,078 (2,245 men/1,833 women); **n = 4,442 (2,337 men/2,105 women). Data are expressed as mean with standard deviation or medians with 1^st^/3^rd^ quartile. BMI stands for body mass index, BP for blood pressure, bpm for beats per minute, FH for family history, CAD for coronary artery disease, MI for myocardial infarction, CHF for congestive heart failure, PAD for peripheral artery disease, CKD for chronic kidney disease, COPD for chronic obstructive pulmonary disease, OC for oral contraceptives, HRT for hormone replacement therapy, FRS for Framingham risk score, SI for stiffness index, CRP for C-reactive protein, WBCC for white blood cells count, IL-18 for interleukin-18, IL-1RA for interleukin-1 receptor antagonist.

As expected, the prevalence of most cardiovascular risk factors (CVRFs) in the present population-based sample was markedly higher among male than in female participants. Stiffness index was 1.7 m/s higher in men compared to women.

Subjects with very stiff vessels were markedly older than subjects with measureable SI, and presented a higher cardiovascular risk profile, being more obese and having more hypertension and diabetes. Moreover, they were at an approx. 3-fold higher risk for future fatal CVD events (i.e.10-year risk for fatal MI or stroke according to the German version of the European Society of Cardiology (ESC) SCORE) and had higher biomarker concentrations in both sexes except for hematocrit (Table 1).

When biomarkers were stratified according to sex-specific tertiles (T) of SI, all circulating markers increased with higher tertiles of SI in both sexes except white blood cell count (WBCC) in women (Table 2).

**Table 2:** Biomarker Distribution According to Tertiles of Stiffness Index (n = 12,650).

Biomarkers	Stiffness index (m/s)		
	Tertile 1*	Tertile 2*	Tertile 3*
Men			
CRP, mg/l	1.2 (0.5/2.4)	1.6 (0.6/3.0)	1.6 (0.7/3.1)
WBCC, 10^9^/L	6.5 (5.5/7.8)	7.0 (5.9/8.4)	6.9 (5.8/8.2)
Neopterin, pmol/l*	5.3 (4.6/6.3)	5.5 (4.7/6.3)	5.6 (4.8/6.6)
IL-18, pg/ml^†^	234 (187/291)	248 (197/322)	249 (195/322)
IL-1RA, pg/ml^‡^	281 (208/366)	321 (248/425)	319 (246/430)
Fibrinogen, mg/dl	299 (260/346)	323 (278/379)	323 (281/374)
Hematocrit, %	43.6 (41.7/45.4)	43.5 (41.7/45.4)	44.0 (42.1/45.8)
Women			
CRP, mg/l	1.4 (0.5/3.0)	1.6 (0.6/3.5)	1.9 (0.9/3.7)
WBCC, 10^9^/L	6.9 (5.8/8.2)	7.1 (5.9/8.3)	6.9 (6.0/8.3)
Neopterin, pmol/l^§^	5.2 (4.7/6.1)	5.3 (4.6/6.3)	5.5 (4.8/6.6)
IL-18, pg/ml^†^	199 (161/245)	205 (165/268)	219 (173/271)
IL-1RA, pg/ml^‡^	310 (234/417)	336 (246/454)	341 (254/455)
Fibrinogen, mg/dl	312 (270/361)	330 (287/382)	335 (292/392)
Hematocrit, %	39.8 (38.0/41.7)	40.2 (38.5/42.0)	40.8 (39.0/42.4)

Data are expressed as medians (1^st^/3^rd^ quartile). P_trend_ for all biomarkers < 0.0001, except WBCC in females with P_trend_ = 0.062. *Tertile cut-points for SI were 6.91 m/s and 9.37 m/s in men, and 5.58 m/s and 7.25 m/s in women. ^§^n = 3,548 (1,886 men/1,662 women); ^†^n = 4,078 (2,245 men/1,833 women); ^‡^n = 4,442 (2,337 men/2,105 women). CRP stands for C-reactive protein, WBCC for white blood cell count, IL-18 for interleukin-18, and IL-1RA for interleukin-1 receptor antagonist.

Overall, linear correlations between biomarkers and SI, however, were very modest; only weak correlations (r between 0.13 and 0.15) were found with fibrinogen in both sexes and interleukin-1 receptor antagonist (IL-1RA) in men and hematocrit in women (see Supplemental Table 1).

Results from multivariable linear regression analysis for SI exploring the association between circulating biomarkers (all per one standard deviation (SD) increase) and AS are shown in Table 3. Although most biomarkers were strongly related to SI in both sexes under adjustment for age, additional controlling for traditional CVRFs, including systolic/diastolic blood pressure and antihypertensive treatment resulted in an attenuation of such associations, which in case of CRP in both sexes and interleukin-18 (IL-18) in men disappeared. No associations were found for neopterin in both sexes, fibrinogen in men and IL-18 in women in any model. Further adjustment for statins and antiplatelet therapy, as well as hormonal influence (in women only), had no impact on calculated estimates (Supplemental Table 2).

**Table 3:** Markers of Inflammation and Hemostasis and Arterial Stiffness: Results of Sex-specific Multivariable Linear Regression for Stiffness Index Among Individuals with Quantifiable Measurement (n = 12,650).

	Adjustment for age	Additional adjustment for cardiovascular risk factors* and blood-pressure lowering medication**		
	β (95% CI)	p value	β (95% CI)	p value
Men				
CRP	0.16 (0.10/0.21)	<0.0001	0.04 (−0.01/0.10)	0.15
WBCC	0.18 (0.13/0.23)	<0.0001	0.08 (0.02/0.13)	0.0064
Neopterin^§^	−0.06 (−0.15/0.03)	0.18	0.01 (−0.08/0.09)	0.90
IL-18^†^	0.11 (0.03/0.19)	0.0085	0.06 (−0.01/0.14)	0.10
IL-1RA^‡^	0.18 (0.10/0.25)	<0.0001	0.11 (0.03/0.18)	0.0096
Fibrinogen	0.05 (−0.01/0.10)	0.11	−0.04 (−0.10/0.01)	0.13
Hematocrit	0.30 (0.25/0.35)	<0.0001	0.16 (0.10/0.21)	<0.0001
Women				
CRP	0.09 (0.05/0.13)	<0.0001	0.03 (−0.01/0.08)	0.14
WBCC	0.12 (0.08/0.16)	<0.0001	0.07 (0.03/0.11)	0.00086
Neopterin^§^	0.01 (−0.06/0.09)	0.77	0.03 (−0.05/0.10)	0.47
IL-18^†^	0.01 (−0.06/0.09)	0.71	−0.01 (−0.08/0.06)	0.83
IL-1RA^‡^	0.09 (0.03/0.16)	0.0057	0.07 (0.00/0.14)	0.039
Fibrinogen	0.10 (0.06/0.15)	<0.0001	0.05 (0.00/0.09)	0.038
Hematocrit	0.22 (0.18/0.26)	<0.0001	0.14 (0.10/0.18)	<0.0001

Data represent β-estimates (per one SD increase in biomarker concentration) with 95% confidence interval. Models were calculated for each biomarker with stiffness index as dependent variable. CRP, IL-18, IL-1RA and neopterin were log-transformed for analysis.

*Systolic and diastolic blood pressure, diabetes mellitus, obesity, smoking, dyslipidemia, family history of MI/stroke; **blood-pressure lowering medication (ATC codes: C02, C03, C07, C08, C09); ^§^n = 3,548 (1,886 men/1,662 women); ^†^n = 4,078 (2,245 men/1,833 women); ^‡^n = 4,442 (2,337 men/2,105 women). CI stands for confidence interval, CRP for C-reactive protein, WBCC for white blood cell count, IL-18 for interleukin-18, and IL-1RA for interleukin-1 receptor antagonist.

Smoking, followed by hypertension were the main factors determining the “biomarker – risk factors” associations, as demonstrated sex-specifically in Supplemental Tables 3 and 4.

**Figure 2:**
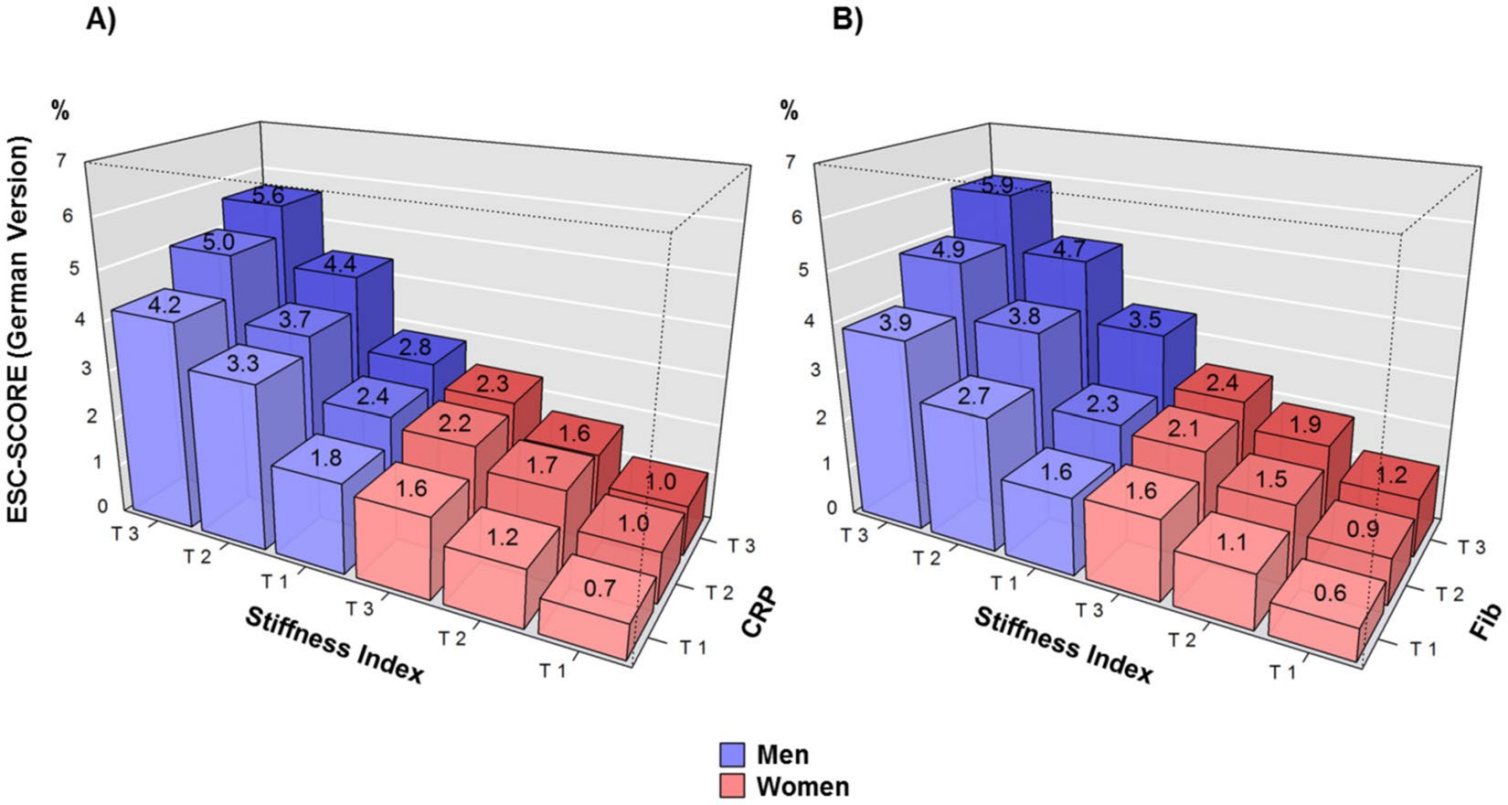
Ten-year Risk for Fatal MI or Stroke According to the German Version of the ESC-SCORE by Tertiles of Stiffness Index and (**A**) C-reactive Protein or (**B**) Fibrinogen. CRP stands for C-reactive protein and Fib for fibrinogen.

In the next step, the combination of both, biomarkers and SI was evaluated for cardiovascular risk assessment. Increasing values of SI and biomarker (both categorized in tertiles) demonstrated an additive effect on the 10-year risk for fatal MI or stroke according to the German version of the ESC-SCORE (Fig. 2; illustrated for CRP and fibrinogen), as well as on the 10-year risk for incident CVD according to the updated Framingham Risk Score (FRS) (Supplemental Fig. 2, again illustrated for CRP and fibrinogen).

**Figure 3:**
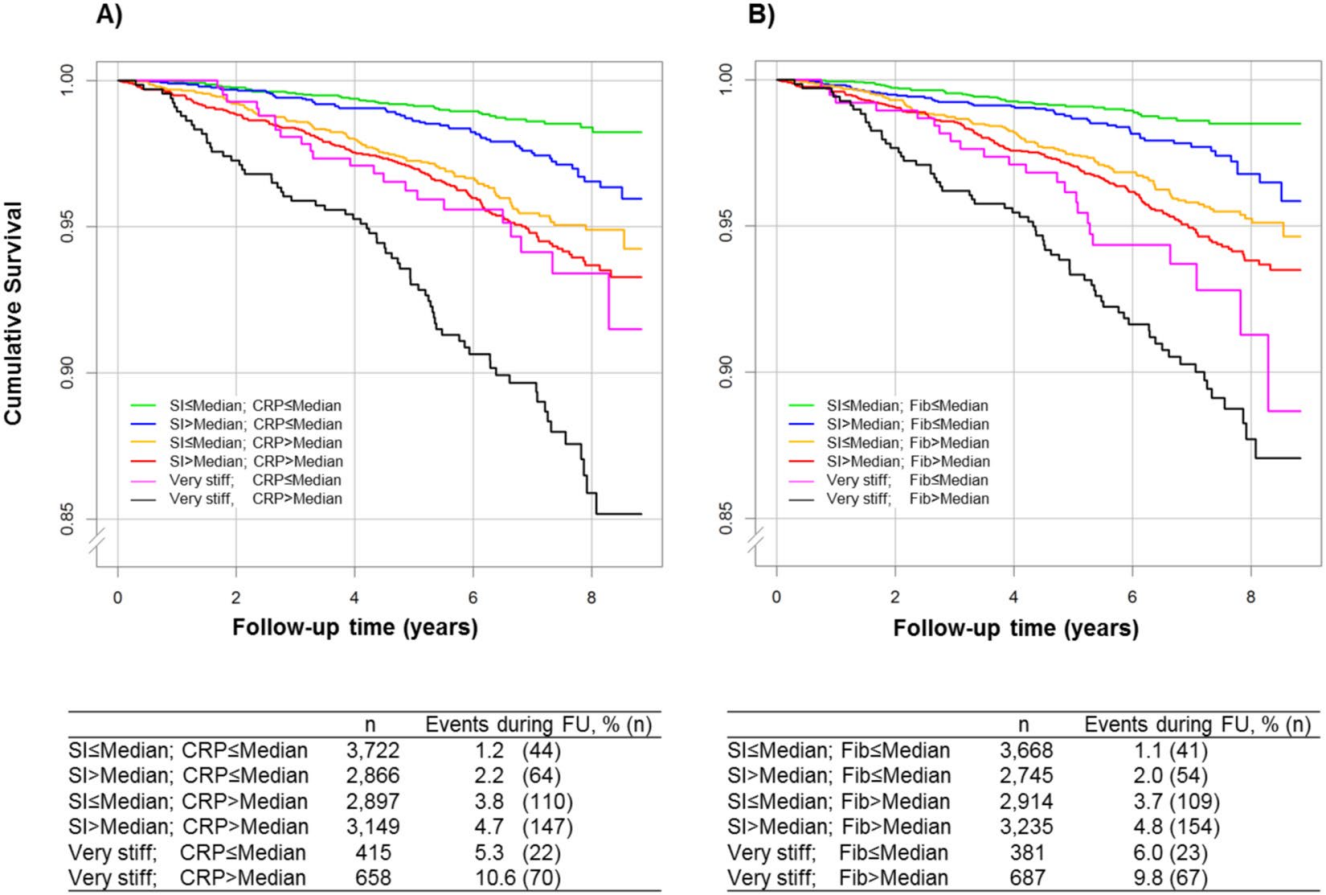
Effect of Arterial Stiffness and (**A**) C-reactive Protein or (**B**) Fibrinogen on Survival Over 8 Years. Panels display Kaplan-Meier Curves for a 8-year follow-up period according to arterial stiffness (with stiffness index below, equal and above the median, or very stiff), and CRP or fibrinogen below or equal and above the median concentration. SI stands for stiffness index, CRP for C-reactive protein and Fib for fibrinogen.

For further evaluation of the prognostic impact on all-cause mortality, SI and each biomarker were dichotomized (below or equal versus above the median concentration). During the follow-up period of 6.2 ± 1.7 years, a total of 511 deaths (332 men/179 women) occurred. Stiffness index was higher at baseline in subjects with an event compared to event-free individuals (7.5 ± 2.2 m/s versus 8.2 ± 2.1 m/s, p< 0.0001). Figure 3 displays Kaplan-Meier curves for CRP and fibrinogen for the endpoint all-cause mortality according to the varying categories of SI and of biomarker concentrations. For all markers, subjects with both markers above the median demonstrated worse survival and with both below the median the best survival except for hematocrit (Supplemental Fig. 3). The combination of very stiff arteries and biomarker concentrations above the median was related to the worst survival (Fig. 3).

**Figure 4:**
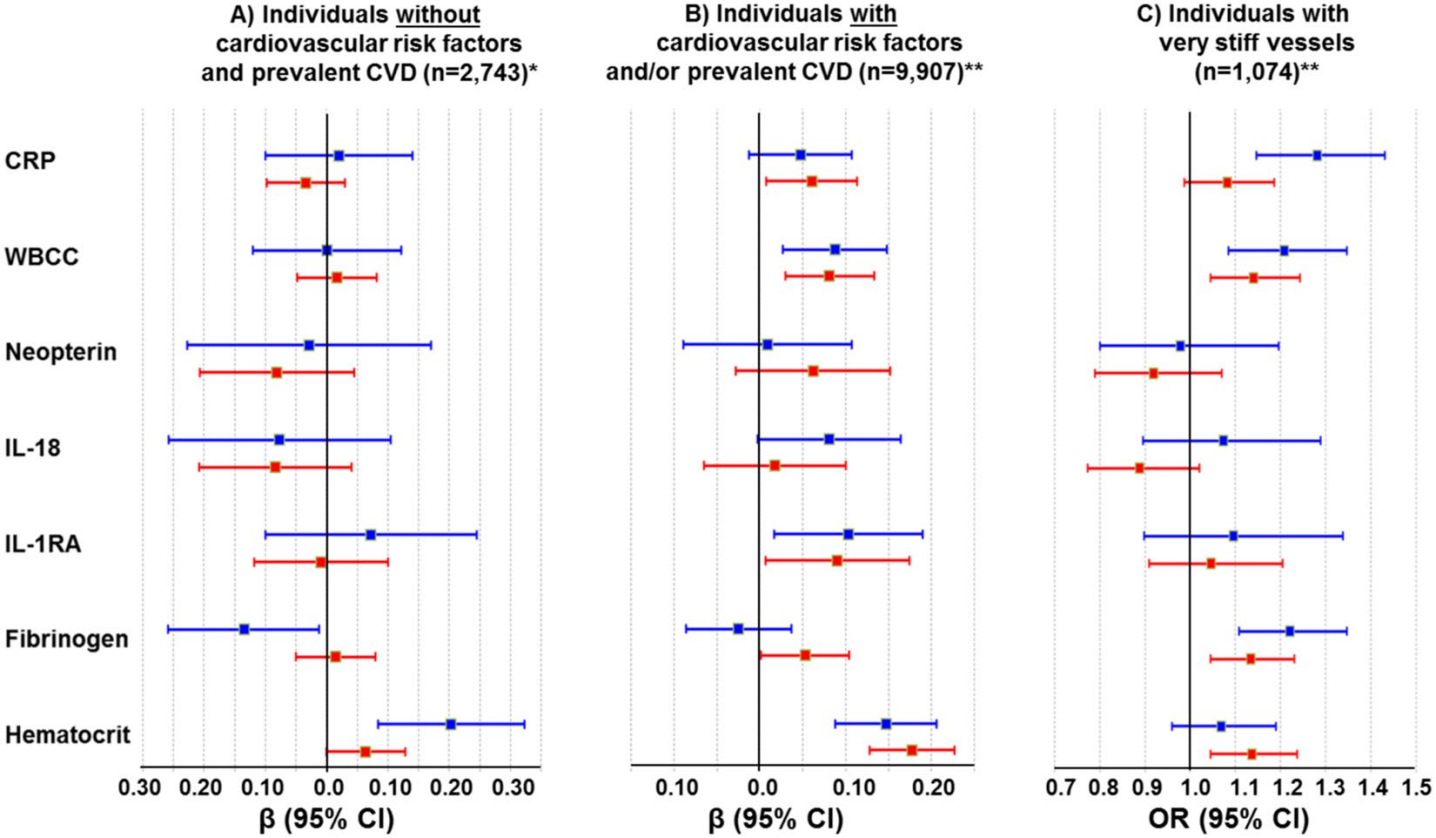
Relation of Stiffness Index with Markers of Inflammation and Hemostasis According to Cardiovascular Risk Profile. ﻿Estimates for men are presented in blue, and for women in red. Linear regression analysis with stiffness index as dependent variable was applied for samples (**A** and **B**). Data represent β-estimates per 1-SD increase in biomarker concentration with 95% CIs. Logistic regression analysis with presence of very stiff vessels as dependent variable (as dichotomous trait: very stiff versus measurable SI) was applied for sample (**C**). Data represent ORs per 1-SD increase in biomarker concentration with their 95% CIs. *Model adjusted only for age, systolic and diastolic blood pressure, since cardiovascular risk factors and cardiovascular disease are not present in this sample. **Models adjusted for age, traditional cardiovascular risk factors (systolic and diastolic blood pressure, diabetes mellitus, obesity, smoking, dyslipidemia, FH on MI/stroke) and blood-pressure lowering medication. OR stands odds ratio, CI for confidence interval, CRP for C-reactive protein, WBCC for white blood cell count, IL-18 for interleukin-18, IL-1RA for interleukin-1 receptor antagonist, and CVD for cardiovascular disease.

For assessing the variation of the association between SI and inflammation according to the cardiovascular risk profile, the individuals from the GHS sample (n = 13,724) were stratified in subjects free of CVRFs and manifest CVD (n = 2,743), those with prevalent CVRFs and/or CVD (n = 9,907) (Supplemental Table 5) and subjects rated as having very stiff vessels (n = 1,074). Interestingly, the only association observed within the physiological milieu (i.e. in absence of CVRFs and CVD) was between SI and hematocrit in males (β = 0.20, p = 0.00085, in men after adjustment for age, SBP and DBP; Fig. 4). With increasing cardiovascular risk, additional associations were found between biomarkers and SI: In individuals with prevalent CVRFs or CVD, an increase in WBCC and, IL-1RA concentration (additionally to hematocrit) was associated with higher SI values in both sexes independently of potential confounders (Fig. 4; model, adjusted for traditional CVRF and BP lowering drugs). In females, an association was also found with CRP and borderline with fibrinogen. Associations were even more pronounced among subjects with very stiff vessels. For instance, each increase in CRP, WBCC or fibrinogen concentrations by one SD in men were associated with a 28%, 21% and 22% increased risk of having a stiff vasculature (Fig. 4; model, adjusted for traditional CVRF and BP lowering drugs). In women, these associations were slightly weaker for WBCC and fibrinogen, but absent for CRP. Surprisingly, IL-1RA in both sexes and hematocrit in men were not related to the presence of very stiff arteries. Again, neither neopterin nor IL-18 was related with SI in these subsamples. Again, further adjustment for statins and antiplatelet therapy, as well as hormonal influence (in women only), had no impact on observed associations (Supplemental Table 2).

## Discussion

Within the present analysis, the clinical relevance of inflammation and hemostasis for AS has been evaluated in the largest population-based investigation to date. To the best of our knowledge this is the first study demonstrating the relation between a panel of biomarkers reflecting these processes and SI derived from digital photoplethysmography.

Strong and independent associations of hematocrit, WBCC and IL-1RA with SI were found among both men and women. Fibrinogen was also related with AS in females. Interestingly, associations between inflammatory and hemostatic markers with SI were significantly influenced by CVRFs, especially by smoking and hypertension. This supports their pathophysiological effects on the arterial tree in cardiovascular or arteriosclerotic disease.

C-reactive protein represents the best studied biomarker of cardiometabolic disorders so far^3, 4, 24^. The most pronounced change was seen for CRP, where the significant association with SI disappeared in the fully adjusted model in men (remaining borderline in women). Functionally, CRP was proposed to have effects that may influence initiation and progression of vascular disease, including mediation of vascular dysfunction, induction of a pro-thrombotic state and cytokine release, activation of the complement system and facilitation of extracellular matrix remodeling^24^. Though, epidemiological data on a cross-sectional relationship between CRP and measures of AS from the literature are controversial^8–10^. Remarkably, CRP was a strong predictor of PWV in a prospective setting within longer follow-up periods of 16^11^ or 20^12^ years, whereas the investigation with a shorter follow-up time^13, 14^ revealed no meaningful relationship of CRP to stiffness measures. This rather suggests a long-term effect of inflammation on AS.

Hematocrit, a well-known predictor of future cardiovascular events^25, 26^, demonstrated the strongest association with SI in all subsamples with measurable stiffness. It was the only marker associated with SI in the physiological state of presumably healthy males. Its important role as determinant of SI is not surprising, since AS is highly related to mechanical property of blood flow^27^. The fact that the well-known correlates of hematocrit such as male sex, smoking and blood pressure^28^ were also major determinants of SI in our previous analysis^22^, supports a possible synergistical effect of this “risk marker - risk factor” clustering.

While the association between fibrinogen and SI found in the present analysis is expected, due to close relation between hematocrit and fibrinogen^28^, the sex difference in this association is remarkable. It was found in females only and remained also after adjusting for hormonal influence, which possibly implies an important role of other conventional CVRFs in vascular stiffening among women. Current published data on the association between fibrinogen and AS are contradictory: no association between fibrinogen and PWV was reported from participants from the Framingham Offspring Study^10^, 429 apparently healthy middle-aged women^15^ or patients with end-stage renal disease^16^. In contrast, a positive association between increased fibrinogen concentrations and cf-PVW was found in 229 hypertensive and 159 age-matched normotensive individuals^17^.

IL-1RA was strongly positively related with SI in both sexes even after adjustment for traditional CVRFs and various medications within the present analysis. This naturally occurring anti-inflammatory antagonist of the interleukin-1 family of pro-inflammatory cytokines is currently also applied in the treatment of various inflammatory conditions^29^. Increased IL-1RA concentrations at baseline were found to be associated with an increased risk for incident CVD and type 2 diabetes mellitus^30^. Only one study has evaluated the role of IL-1RA in decreased arterial compliance so far and identified IL-1RA as a strong predictor of aortic PWV over 16 years^11^. The non-inverse association between IL-1RA and SI supports the idea of understanding this marker as counter-regulated response to interleukin 1β (IL-1β)-mediated pro-inflammatory stimuli^30^. Currently, IL-1β represents one of the substantive upstream inflammatory cytokine and major target for immunomodulation, since its inhibition has already been linked to the reduction in IL-6, CRP and fibrinogen concentration in the high vascular risk patients^31^. Interestingly, although IL-1β and IL-18 belong to the same IL-1 family of cytokines and shear similar regulation by NLRP3 inflammasome^32^ they seem have differential impact on vasculature within the present analysis. IL-18 is considered as a potent inducer of interferon-γ (INF-γ) production, thereby resulting in the enhanced expression of matrix metalloproteinases, and subsequent degeneration of compliant elastin fibers^33, 34^. Several differences in IL-1β and IL-18 signaling have been already reported^35^, with a potent activation of nuclear factor-kappa B by IL-1β and only weak or even null effects on it in case of IL-18^36^. In distinction, IL-18/IFN-γ effects are mediated mainly through activation of JAK-STAT pathway^37^. Moreover, to ensure adequate cell activation, IL-18 requires a co-stimulant (most commonly IL-12) as well as the much higher concentration than IL-1β, which, in contrast, is already active in the low picomol range^33, 35^. Other potential mechanism for the observed discrepancies might include induction of cyclooxygenase-2 expression by IL-1β with subsequent production of prostaglandin E_2_^33, 35^, as a key mediator of vascular remodeling and negative regulator of IFN-γ-mediated response. On the contrary, neither cyclooxygenase-2 nor other acute phase proteins such as e.g. IL-6 could be sufficiently induced by IL-18^33, 35^. Thus, one might speculate that other than IFN-γ-mediated effects might be detrimental for the relationship between inflammation and AS. Further indirect confirmation of this represents a fact, that neopterin, as a protein releasing upon stimulation with IFN-γ, was also not associated with SI in the current study. In addition to being a marker of IFN-γ inducible inflammation, neopterin possesses potent pro-oxidative properties that might be responsible for destabilization and vulnerability of the arterial wall^37^. Despite their hypothetical crucial role in AS, neither IL-18 nor neopterin presented as independent determinant of SI.

**Figure 5:**
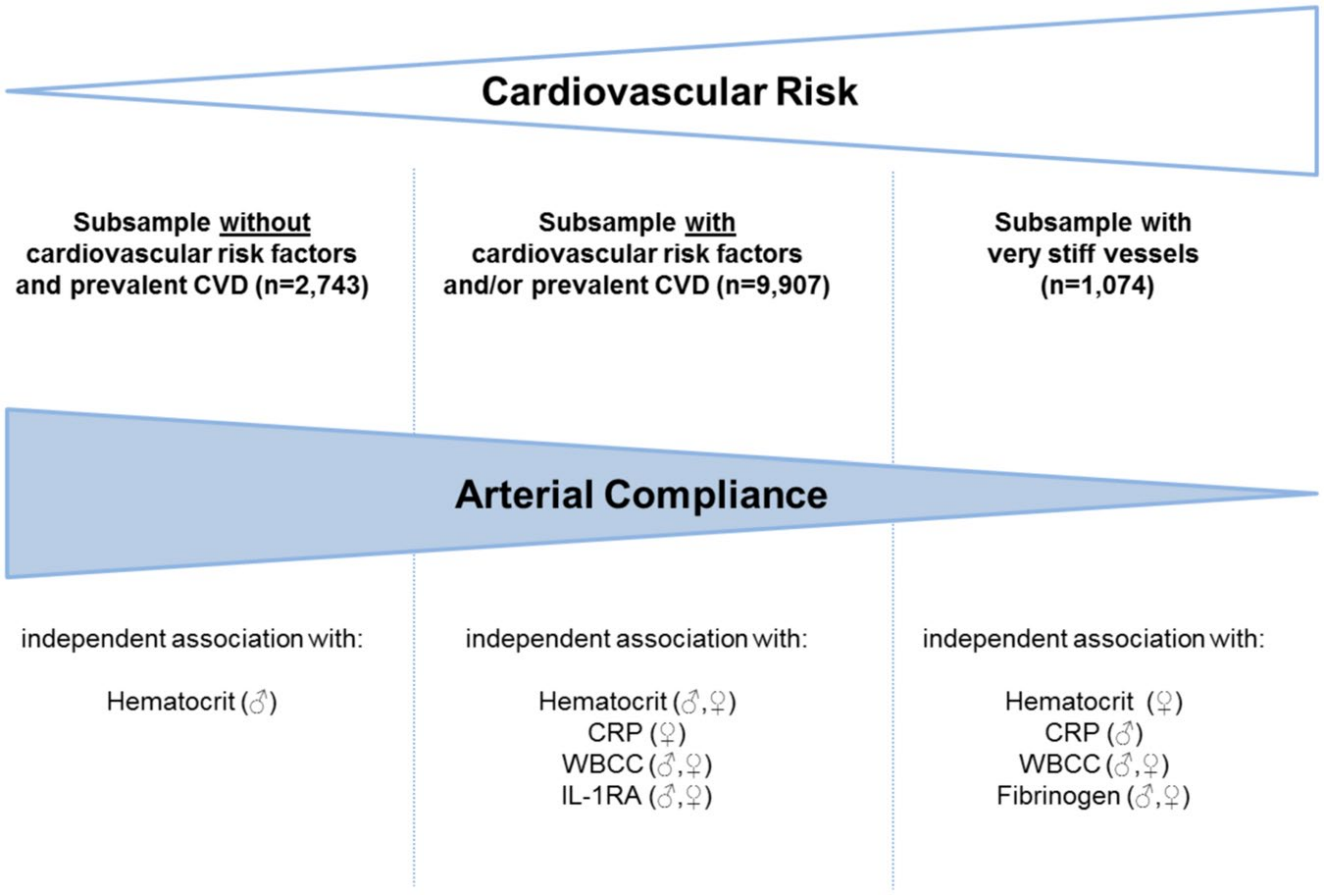
Differential Impact of Markers of Inflammation and Hemostasis on Arterial Compliance According to Cardiovascular Risk Profile. CVD stands for cardiovascular disease, CRP for C-reactive protein, WBCC for white blood cell count, and IL-1RA for interleukin-1 receptor antagonist.

Interestingly, the patterns of association between circulating biomarkers and AS were varying from low to higher cardiovascular risk profiles (summarized in Fig. 5). The differential associations with SI in the range from presumably cardiovascular healthy individuals, via those with CVRFs or established disease to individuals with severe AS (i.e. “very stiff” status) indicate a variable significance of biomarkers in the development and progression of AS and the state, where arterial compliance is completely lost.

The present analysis has several limitations which need to be considered: all biomarkers were measured only once in this large sample, therefore data could include a regression dilution bias. Although biomarker concentrations mediated the relation between SI with future cardiovascular risk as indicated by risk scores, no direct evidence was investigated for this effect. Currently published evidences on SI (clinical, epidemiological etc.) are not as widely available compared to the methods, assessing e.g. stiffness of the large arteries, such as carotid-femoral pulse wave velocity (cf-PWV). Finally, results may not be extrapolated to other populations, ethnicities or age groups.

The strengths of this study are the investigated well-characterized, population-representative, large-scale cohort sample with sufficient enough power to assess associations sex-specifically. Various traditional CVRFs were carefully assessed by measurements and not based on self-reported data only, which minimizes a possible misclassification. In contrast to other studies, several inflammatory and hemostatic markers were analyzed simultaneously.

The present analysis demonstrated a direct relationship of inflammation and hemostasis with SI measured by digital photoplethysmography, which was modulated by the extent of arterial stiffening. Simultaneous evaluation of biomarkers and SI improved the assessment of the risk for future CV events or mortality. The mediation of the relation between chronic low-grade inflammation and SI by smoking and arterial hypertension supports the idea of nonpharmacological (e.g. smoking cessation) or pharmacological (e.g. appropriate antihypertensive treatment or hypothetically even anti-inflammatory agents, such as e.g. canakinumab) interventions for improvement of arterial compliance.

## Methods

### Study Design, Population and Follow-up Investigation

The GHS is a population-based, prospective, observational, single-center cohort study, including residents of the City of Mainz and the County Mainz-Bingen from Western Germany. Details of the study design have been reported elsewhere^38^. Briefly, individuals between 35 and 74 years of age were randomly selected from the local registration offices. The sample was stratified 1:1 for sex and present residence (urban/rural) with equal strata for decades of age. Exclusion criteria were insufficient knowledge of the German language and physical or mental inability to participate in the examinations. Participation was voluntary and written informed consent was obtained from each subject upon entry into the study. The study conduct is performed according to the principles of good clinical practice and the principles outlined in the Declaration of Helsinki. The study protocol, study documents and sampling design were approved by the local ethics committee (medical association of the federal state Rhineland-Palatinate, Germany (reference number 837.020.07(5555)) and the data safety commissioner of the University Medical Center of the Johannes Gutenberg University Mainz, as well as by the steering committee of the Gutenberg Health Study.

The present analysis is based on baseline data of 15,010 GHS-participants enrolled from April 2007 to April 2012. The outcome investigated in the present analysis was all-cause mortality. Death was ascertained by the respective entry in the registry office and the death certificate. For all 511 subjects (332 men/179 women), who died during follow-up complete information on all variables of interest was available.

### Assessment of Arterial Stiffness

Arterial stiffness was assessed using a Pulse Trace PCA2 device (Micro Medical Limited/Carefusion). This method is based on digital photoplethysmography, which transmits an infrared light at 940 nm through the finger with the amount of absorbed light being proportional to the volume of blood in the finger pulp. The plethysmography transducer, a non-invasive finger clip, was placed on the subject’s ring (or fourth) finger and 10 pulses were recorded to produce a representative pulse waveform. This waveform consists of an early systolic and a second diastolic peak. The time difference between these two peaks (so called “peak-to-peak time”, PPT) was used to calculate the SI, defined as the subjects height in meter divided by PPT in seconds (Supplemental Fig. 1A). In subjects, where a discrimination between systolic and diastolic peaks on digital pulse curve was not feasible (i.e. no distinct notch in the waveform, defined as class 4 waveform according to Dawber *et al*.^23^), AS was categorized as “very stiff” (Supplemental Fig. 1B). All measurements were performed according to standard operating procedures with calibration for the device used and detailed quality control.

### Data Collection, Definitions of Cardiovascular Risk Factors and Laboratory Methods

For additional details and information on laboratory methods please see the Supplementary Description of Methods.

### Statistical Analysis

Analyses were carried out sex-specifically. Concentrations of biomarkers were reported as median with interquartile range or as mean with standard deviation (SD), where appropriate. The circulating biomarkers were further stratified into tertiles and Mann-Whitney U-test was used to analyze group differences. Correlations between biomarkers and SI were assessed by Pearson correlation coefficients.

Linear regression analysis was performed to assess the relationship between SI and biomarkers (per one SD increase) with a logarithmic transformation of skewed variables in the model (CRP, neopterin, IL-18, IL-1RA). The basic model was adjusted for age; further analyses were adjusted for traditional CVRFs such as systolic/diastolic BP, obesity, dyslipidemia, smoking, diabetes and family history of myocardial infarction (MI) or stroke and blood-pressure lowering medication (Anatomical Therapeutic Chemical (ATC) codes: C02, C03, C07, C08, C09), and additionally (in Model 3) for statin intake, antiplatelet therapy, and in females for menopausal status, and intake of oral contraceptives (OC) or hormone replacement therapy (HRT). Results are reported as β-estimates with 95% confidence intervals (CIs). Absolute and relative differences in β-estimates between crude and CVRFs-adjusted model were determined to evaluate the impact of CVRFs on “SI-biomarker” association. The potential additive effect of SI and biomarkers on the 10-year CVD risk was evaluated for the German version of the ESC SCORE and the updated Framingham risk score^39, 40^. Kaplan–Meier survival analysis was performed to evaluate the combined effect of AS (SI or presence of very stiff vessels) and biomarkers on all-cause mortality. Differences in Kaplan–Meier mortality curves were assessed using the log-rank test.

Finally, to evaluate the association between SI and biomarkers in dependence on cardiovascular risk, the study sample was stratified into three subgroups: Individuals without CVRF and established CVD (MI, coronary artery disease (CAD), congestive heart failure (CHF) or stroke) (n = 2,743) served as group with low risk; individuals with CVRFs and/or CVD (n = 9,907) as group with intermediate risk and subjects with very stiff vessels as an equivalent of a cardiovascular high risk group (n = 1,074). In subjects with very stiff vessels, multivariable logistic regression was applied (dependent variable was treated dichotomously as very stiff versus measurable SI). Results are reported as β-estimates or odds ratio (OR) with 95% CIs, where appropriate.

Because of the explorative character of the analysis, a significance threshold was not defined for p-values. P values should be interpreted as a continuous measure of statistical evidence. All statistical analyses were performed using R version 3.14.2 software (http://www.r-project.org).

## Electronic supplementary material

Supplementary file

## References

[1] Puddu PE (2011). Determinants of 40-year all-cause mortality in the European cohorts of the Seven Countries Study. Eur. J. Epidemiol..

[2] Puddu PE, Schiariti M, Torromeo C (2016). Gender and cardiovascular mortality in Northern and Southern European Populations. Curr. Pharm. Des..

[3] Sesso HD (2003). C-reactive protein and the risk of developing hypertension. JAMA..

[4] McEniery CM, Wilkinson IB (2005). Large artery stiffness and inflammation. J. Hum. Hypertens..

[5] Flamant M (2007). Role of matrix metalloproteinases in early hypertensive vascular remodeling. Hypertension..

[6] Wu J (2016). Immune activation caused by vascular oxidation promotes fibrosis and hypertension. J. Clin. Invest..

[7] Floege J, Ketteler M (2004). Vascular calcification in patients with end-stage renal disease. Nephrol. Dial. Transplant..

[8] Schnabel R (2008). Relations of inflammatory biomarkers and common genetic variants with arterial stiffness and wave reflection. Hypertension..

[9] Yasmin (2004). C-reactive protein is associated with arterial stiffness in apparently healthy individuals. Arterioscler. Thromb. Vasc. Biol..

[10] Lieb W (2009). Multimarker approach to evaluate correlates of vascular stiffness: the Framingham Heart Study. Circulation..

[11] Johansen NB (2012). Determinants of aortic stiffness: 16-year follow-up of the Whitehall II study. PLoS. One..

[12] McEniery CM (2010). An analysis of prospective risk factors for aortic stiffness in men: 20-year follow-up from the Caerphilly prospective study. Hypertension..

[13] van Bussel BC (2011). Endothelial dysfunction and low-grade inflammation are associated with greater arterial stiffness over a 6-year period. Hypertension..

[14] Tomiyama H (2010). Continuous smoking and progression of arterial stiffening: a prospective study. J. Am. Coll. Cardiol..

[15] Taquet A (1993). Relations of cardiovascular risk factors to aortic pulse wave velocity in asymptomatic middle-aged women. Eur. J. Epidemiol..

[16] Blacher J (1998). Influence of biochemical alterations on arterial stiffness in patients with end-stage renal disease. Arterioscler. Thromb. Vasc. Biol..

[17] Vlachopoulos C (2007). Relationship of fibrinogen with arterial stiffness and wave reflections. J. Hypertens..

[18] Laurent S (2006). Expert consensus document on arterial stiffness: methodological issues and clinical applications. Eur. Heart. J..

[19] Millasseau SC (2006). Contour analysis of the photoplethysmographic pulse measured at the finger. J. Hypertens..

[20] Woodman RJ (2003). Interpretation of the digital volume pulse: its relationship with large and small artery compliance. Clin. Sci. (Lond).

[21] Epstein S (2014). Numerical assessment of the stiffness index. Conf. Proc. IEEE Eng. Med. Biol. Soc..

[22] Khuseyinova N (2012). Age- and gender-specific distribution of arterial stiffness in the population and its association with global cardiovascular risk: results from the population-based Gutenberg Health study. Eur. Heart. J..

[23] Dawber TR, Thomas HE, McNamara PM (1973). Characteristics of the dicrotic notch of the arterial pulse wave in coronary heart disease. Angiology..

[24] Khuseyinova, N. & Koenig, W. C-reactive protein and other inflammatory markers in cardiovascular disease in *Therapeutic Lipidology* (Davidson, M. H., Toth, P. P., Maki, K. eds) 69–112 (Humana Press, 2007).

[25] Danesh J (2000). Haematocrit, viscosity, erythrocyte sedimentation rate: meta-analyses of prospective studies of coronary heart disease. Eur. Heart. J..

[26] Puddu PE (2002). Red blood cells count in short-term prediction of cardiovascular disease incidence in the Gubbio population Study. Acta. Cardiol..

[27] Nichols, W. W., O’Rourke, M. F. & Vlachopoulos, C. The nature of flow of a liquid in *McDonald*’*s blood flow in arteries*: *theoretical*, *experimental and clinical principles*. (Reneman, R. S. ed) 13–54 (Hodder Arnold, 2011).

[28] Lowe GD (1999). Rheological influences on thrombosis. Baillieres. Best. Pract. Res. Clin. Haematol..

[29] Krishnan SM (2014). IL-1β and IL-18: inflammatory markers or mediators of hypertension?. Br. J. Pharmacol..

[30] Herder C (2015). The IL-1 pathway in type 2 diabetes and cardiovascular complications. Trends. Endocrinol. Metab..

[31] Ridker PM (2012). Effects of interleukin-1beta inhibition with canakinumab on hemoglobin A1c, lipids, C-reactive protein, interleukin-6, and fibrinogen: a phase IIb randomized, placebo-controlled trial. Circulation..

[32] Lukens JR, Gross JM, Kanneganti TD (2012). IL-1 family cytokines trigger sterile inflammatory disease. Front. Immunol.

[33] Dinarello CA (2013). Interleukin-18 and IL-18 binding protein. Front. Immunol..

[34] Ishida Y (2004). The role of IL-18 in the modulation of matrix metalloproteinases and migration of human natural killer (NK) cells. FEBS. Letters..

[35] Lee JK (2004). Differences in signaling pathways by IL-1beta and IL-18. Proc. Natl. Acad. Sci. USA.

[36] Voloshyna I, Littlefield MJ, Reiss AB (2014). Atherosclerosis and interferon-γ: new insights and therapeutic targets. Trends. Cardiovasc. Med..

[37] Fuchs D (2009). The role of neopterin in atherogenesis and cardiovascular risk assessment. Curr. Med. Chem..

[38] Wild PS (2012). [The Gutenberg Health Study]. Bundesgesundheitsblatt. Gesundheitsforschung. Gesundheitsschutz..

[39] Keil U (2005). Risk stratification with the new ESC risk tables for Germany. Herz..

[40] D’Agostino RB (2008). General cardiovascular risk profile for use in primary care: The Framingham heart study. Circulation..

