# Supplementary material for: Relation between Arterial Stiffness and Markers of Inflammation and Hemostasis – Data from the Population-based Gutenberg Health Study
Source: Sci Rep. 2017 Jul 24;7:6346. doi: 10.1038/s41598-017-06175-2 (PMC5524791; doi:10.1038/s41598-017-06175-2)
Supplement: Supplementary file 1 — Supplementary file [file 41598_2017_6175_MOESM1_ESM.docx]

**SUPPLEMENT**

**Relation between Arterial Stiffness and Markers of Inflammation and Hemostasis – Data from the Population-based Gutenberg Health Study**

Natalie Arnold^1,2,3^, Tommaso Gori^1,3,4,^, Renate B. Schnabel^5,6^, Andreas Schulz^2,3^, Jürgen H. Prochaska^1,3,7^, Tanja Zeller^5,6^, Harald Binder^3,8^, Norbert Pfeiffer^3,9^, Manfred Beutel^3,10^, Christine Espinola-Klein^1,3^, Karl J. Lackner^3,11^, Stefan Blankenberg^5,6^,

Thomas Münzel^1,3,4,7^, and Philipp S. Wild ^2,3,4,7*^

^1^Center for Cardiology I, Center for Cardiology, University Medical Center of the Johannes Gutenberg-University Mainz, Mainz, Germany; ^2^Preventive Cardiology and Preventive Medicine, Center for Cardiology, University Medical Center of the Johannes Gutenberg-University Mainz, Mainz, Germany; ^3^Center for Translational Vascular Biology (CTVB), University Medical Center of the Johannes Gutenberg-University Mainz, Mainz, Germany; ^4^DZHK (German Center for Cardiovascular Research), Partner Site RhineMain, Mainz, Germany; ^5^Department of General and Interventional Cardiology, University Medical Center Hamburg-Eppendorf, Hamburg, Germany; ^6^DZHK (German Center for Cardiovascular Research), Partner Site Hamburg/Kiel/Lübeck, Hamburg, Germany; ^7^Center for Thrombosis and Hemostasis, University Medical Center of the Johannes-Gutenberg University Mainz, Mainz, Germany; ^8^Institute of Medical Biostatistics, Epidemiology and Informatics (IMBEI), University Medical Center of the Johannes Gutenberg-University Mainz, Mainz, Germany; ^9^Department of Ophthalmology, University Medical Center of the Johannes Gutenberg-University Mainz, Mainz, Germany; ^10^Department of Psychosomatic Medicine and Psychotherapy, University Medical Center of the Johannes Gutenberg-University Mainz, Mainz, Germany; ^11^Institute for Clinical Chemistry and Laboratory Medicine of the Johannes Gutenberg-University Mainz, Mainz, Germany.

***Corresponding Author**

Philipp S. Wild, MD, MSc

Professor for Clinical Epidemiology

Preventive Cardiology and Preventive Medicine

Center for Cardiology,

University Medical Center of the Johannes Gutenberg-University Mainz

Langenbeckstr 1

55131 Mainz, Germany

Telefon  +49 (0) 6131 17-7163 
Telefax  +49 (0) 6131 17-8460
E-Mail:  philipp.wild@unimedizin-mainz.de

**Part A.** Supplemental Methods

**Part B.** Supplemental Tables

**Part C:** Supplemental Figures

**Part A. SUPPLEMENTAL METHODS**

**Data Collection and Definition of Cardiovascular Risk Factors and Diseases**

All study participants underwent a standardized computer-assisted personal interview carried out by a specifically trained team of interviewers. Moreover, all subjects participated in a 5-hour baseline-examination at the study center, which was performed according to standard operating procedures by certified medical technical assistants. All measurement procedures have been described in detail elsewhere^1-2^. Hypertension was diagnosed, if antihypertensive drugs were taken, or a mean examination systolic blood pressure of ≥140mmHg or a mean diastolic blood pressure of ≥90mmHg (averaging the 2^nd^ and 3^rd^ standardized measurements after 8 and 11 minutes of rest). Body mass index (BMI) was calculated as weight in kilograms divided by height in meters squared (both measured). Obesity was defined as a BMI ≥30 kg/m^2^. Smoking behavior was dichotomized into non-smoking (never or former smokers) and smoking (occasional and daily smokers). Subjects with a LDL/HDL-ratio of >3.5 or with diagnosis of dyslipidemia by general practitioners were classified as having dyslipidemia. A positive family history of myocardial infarction or stroke was recorded in a female first-degree relative ≤65 years or in a male first-degree relative ≤60 years. Diabetes mellitus was defined by already diagnosed or by a blood glucose level of ≥126 mg/dl or HbA1c of >6.5 % at the baseline examination after an overnight fast of at least 8 hours, or a blood glucose level of ≥200mg/dl in the baseline examination after a fasting period <8 hours. In addition, those who were on oral blood glucose–lowering therapy or on insulin substitution were also classified as diabetics. Cardiovascular diseases were documented in a computer assisted personal interview by specifically trained and certified interviewers. Participants were asked to bring their medical records and reports to the interview. A disease was recorded as present, if a physician had diagnosed the disease.

**Laboratory Methods**

Venous blood was drawn under standardized conditions from all study participants after an overnight fasting period. All samples were stored at -80°C until further analysis. No samples were inadvertently thawed during storage.

C-reactive protein (CRP) concentration was measured in plasma by latex-enhanced immunoturbidimetric analysis on Architect *c*8000 analyzer (Abbott Laboratories, Abbott Park, Illinois). The limit of detection was ≤0.1 mg/L for the ultrasensitive calibrator and ≤0.2 mg/L for the wide-range calibrator. Fibrinogen was determined by derived method (Siemens; lower detection limit40 mg/dl). Albumin, white blood cells count and hematocrit were done by routine methods. Interleukin-18 (IL-18) and interleukin-1 receptor antagonist (IL1-RA) were determined in serum of 4,078 and 4,442 participants respectively, by commercial available ELISAs: IL1-RA (Quantikine, R&D Systems, Wiesbaden, Germany; minimum detection limit 31.2 pg/mL); IL-18 (Human IL-18 ELISA Kit, MBL, Woburn, MA, USA, minimum detection limit 128 pg/ml). Neopterin was assessed in 3,548 participants by ELISA Neopterin (B.R.A.H.M.S). The intra-assay coefficients of variation (CVs) for CRP was 1.1% to 3.1%; for WBCC 2.95% to 3.0%; for albumin 0.1%; for hematocrit 0.75% to 0.78%; for fibrinogen 1.8% to 2.1; for IL-1RA 11.9%; for IL-18 7.1% and for neopterin 3.0 %. The corresponding values for the inter-assay CVs were for WBCC 1.6% to 3.6%; for albumin 1.2% to 1.3%; for hematocrit 1.8% to 2.0%; for fibrinogen 4.2% to 4.7; for IL-1RA 23.1%; for IL-18 19.2%; for neopterin 6.57 % (3). All analyses were run in a blinded fashion.

**REFERENCES**

1. Wild, P.S. et al. Distribution and categorization of left ventricular measurements in the general population: results from the population-based Gutenberg Heart Study. *Circ. Cardiovasc. Imaging.* **3**:604-13 (2010).
2. Grossmann, V. et al. Profile of the immune and inflammatory response in individuals with prediabetes and type 2 diabetes. *Diabetes. Care.* **38**:1356-1364 (2015).
3. Schnabel, R.B. et al. Multiple endothelial biomarkers and noninvasive vascular function in the general population: the Gutenberg Health Study. *Hypertension.* **60**:288-95 (2012).

**Part B. SUPPLEMENTAL TABLES**

**Supplemental Table 1. Pearson Correlation Coefficients Between Stiffness Index and Markers of Inflammation and Hemostasis**

| **Biomarker** | **Pearson Correlation Coefficients** | |
| --- | --- | --- |
|  | **Men** | **Women** |
| CRP | 0.04 | 0.03 |
| WBCC | 0.08 | 0.03 |
| Neopterin * | 0.03 | 0.07 |
| IL-18 ^†^ | 0.08 | 0.05 |
| IL-1RA^‡^ | 0.13 | 0.06 |
| Fibrinogen | 0.14 | 0.15 |
| Hematocrit | 0.08 | 0.14 |

CRP stands for C-reactive protein, WBCC for white blood cell count, IL-18 for interleukin-18, and IL-1RA=interleukin-1 receptor antagonist.

^*^n=3,548 (1,886 men/1,662 women); ^†^n=4,078 (2,245 men/1,833 women); ^‡^n=4,442 (2,337 men/2,105 women).

**Supplemental Table 2: Markers of Inflammation and Hemostasis and Arterial Stiffness: Results of Fully Adjusted * Sex-specific Multivariable Linear Regression**

|  | 1. Individuals with quantifiable SI measurement (n=12,650) | | 1. Individuals without cardiovascular risk factors and prevalent CVD (n=2,743) | | 1. Individuals with cardiovascular risk factors and prevalent CVD (n=9,907) | | 1. Individuals with very stiff vessels (n=1,074) | |
| --- | --- | --- | --- | --- | --- | --- | --- | --- |
|  | β (95% CI) | p value | β (95% CI) | p value | β (95% CI) | p value | OR (95% CI) | p value |
| **Men** |  |  |  |  |  |  |  |  |
| CRP | 0.04 (-0.01/0.10) | 0.15 | 0.01 (-0.11/0.13) | 0.89 | 0.05 (-0.01/0.11) | 0.12 | 1.30 (1.16/1.45) | <0.0001 |
| WBCC | 0.08 (0.02/0.13) | 0.0064 | 0.005 (-0.12/0.12) | 0.94 | 0.09 (0.03/0.15) | 0.0043 | 1.21 (1.08/1.34) | 0.00069 |
| Neopterin^§^ | 0.01 (-0.08/0.09) | 0.90 | -0.04 (-0.24/0.16) | 0.68 | 0.01 (-0.09/0.11) | 0.83 | 0.99 (0.81/1.21) | 0.92 |
| IL-18^†^ | 0.06 (-0.01/0.14) | 0.10 | -0.08 (-0.27/0.10) | 0.36 | 0.08 (-0.001/0.17) | 0.050 | 1.08 (0.90/1.30) | 0.39 |
| IL-1RA^‡^ | 0.11 (0.03/0.18) | 0.0096 | 0.08 (-0.09/0.25) | 0.37 | 0.10 (0.02/0.19) | 0.020 | 1.11 (0.91/1.36) | 0.31 |
| Fibrinogen | -0.04 (-0.10/0.01) | 0.13 | -0.14 (-0.27/-0.02) | 0.027 | -0.03 (-0.09/0.04) | 0.40 | 1.23 (1.11/1.35) | <0.0001 |
| Hematocrit | 0.16 (0.10/0.21) | <0.0001 | 0.21 (0.09/0.33) | 0.00083 | 0.15 (0.09/0.21) | <0.0001 | 1.07 (0.96/1.19) | 0.21 |
| **Women** |  |  |  |  |  |  |  |  |
| CRP | 0.03 (-0.01/0.08) | 0.14 | -0.04 (-0.10/0.03) | 0.30 | 0.06 (0.01/0.11) | 0.030 | 1.09 (0.99/1.19) | 0.085 |
| WBCC | 0.07 (0.03/0.11) | 0.00086 | 0.02(-0.04/0.09) | 0.55 | 0.09 (0.04/0.14) | 0.00074 | 1.14 (1.05/1.25) | 0.0025 |
| Neopterin^§^ | 0.03 (-0.05/0.10) | 0.47 | -0.08 (-0.21/0.05) | 0.22 | 0.07 (-0.02/0.16) | 0.12 | 0.93 (0.79/1.08) | 0.32 |
| IL-18^†^ | -0.01 (-0.08/0.06) | 0.83 | -0.08 (-0.21/0.04) | 0.19 | 0.01 (-0.07/0.09) | 0.79 | 0.89 (0.77/1.02) | 0.091 |
| IL-1RA^‡^ | 0.07 (0.004/0.14) | 0.039 | -0.01 (-0.12/0.10) | 0.87 | 0.10 (0.01/0.18) | 0.023 | 1.05 (0.91/1.21) | 0.49 |
| Fibrinogen | 0.05 (0.003/0.09) | 0.038 | 0.02 (-0.05/0.09) | 0.54 | 0.05 (0.001/0.10) | 0.046 | 1.14 (1.05/1.24) | 0.0014 |
| Hematocrit | 0.14 (0.10/0.18) | <0.0001 | 0.05 (-0.01/0.12) | 0.12 | 0.17 (0.12/0.22) | <0.0001 | 1.13 (1.04/1.23) | 0.0042 |

Linear regression analysis with stiffness index as dependent variable was applied for samples A, B and C. Data represent β-estimates for stiffness Index per 1-SD increase in biomarker concentration with 95% CIs. Logistic regression analysis with presence of very stiff vessels as dependent variable (as dichotomous trait: very stiff versus measurable SI) was applied for sample D. Data represent ORs per 1-SD increase in biomarker concentration with their 95% CIs. Samples A, C and D: Models were adjusted for age, traditional cardiovascular risk factors (systolic and diastolic blood pressure, diabetes mellitus, obesity, smoking, dyslipidemia, FH on MI/stroke), antihypertensive treatment, statin intake, antiplatelet therapy, and in females additionally adjusted for OC/HRT intake and menopausal status); Samples B: Model was adjusted for age, systolic and diastolic blood pressure, statin intake, antiplatelet therapy, and in females additionally adjusted for OC/HRT intake and menopausal status.

^§^n = 3,548 (1,886 men/1,662 women); ^†^n = 4,078 (2,245 men/1,833 women); ^‡^n = 4,442 (2,337 men/2,105 women).

CI stands for confidence interval, OR stands odds ratio, CRP for C-reactive protein, WBCC for white blood cell count, IL-18 for interleukin-18, and IL-1RA for interleukin-1 receptor antagonist and CVD for cardiovascular disease.

**Supplemental Table 3. Influence of Cardiovascular Risk Factors on the Association Between Stiffness Index and Circulating Biomarkers in Men**

|  | **β-Estimate** | | **Difference in β-Estimates (M1 vs M2)** | | **Influence of CVRF on the association between SI and BM**  **(Absolute Difference in β-Estimate per Risk Factor, M1 vs M2)** | | | | | |
| --- | --- | --- | --- | --- | --- | --- | --- | --- | --- | --- |
|  | Model 1 (M1) | Model 2  (M2) | Absolute | Relative,% | 1  (Strongest) | 2 | 3 | 4 | 5 | 6  (Weakest) |
| CRP | 0.156 | 0.0491 | -0.107 | -68.5 | Smoking  (-0.053) | Obesity  ( -0.024) | Hypertension  (-0.018) | Dyslipidemia  (-0.0067) | Diabetes (0.0026) | FH of MI/stroke (0.00015) |
| WBCC | 0.180 | 0.0542 | -0.125 | -69.8 | Smoking  (-0.093) | Hypertension  (-0.018) | Obesity  (-0.011) | Diabetes  (0.0086) | Dyslipidemia  (-0.0057) | FH of MI/stroke  (-0.0014) |
| Neopterin | -0.0116 | -0.00658 | 0.00506 | -43.5 | Diabetes  (0.0033) | Smoking (0.0014) | Obesity (0.00076) | FH of MI/stroke (0.00069) | Hypertension  (-0.00062) | Dyslipidemia  (0.000085) |
| IL-18 | 0.106 | 0.0687 | -0.0371 | -35.1 | Smoking  (-0.024) | Hypertension  (-0.0072) | Diabetes (0.0061) | Obesity  (-0.0059) | Dyslipidemia  (0.00043) | FH of MI/stroke (0.000078) |
| IL-1RA | 0.176 | 0.0961 | -0.0802 | -45.5 | Smoking  (-0.035) | Obesity  (-0.019) | Hypertension  (-0.018) | Diabetes (0.015) | Dyslipidemia  (-0.014) | FH of MI/stroke (0.00097) |
| Fibrinogen | 0.0453 | -0.0663 | -0.112 | -246 | Smoking  (-0.070) | Hypertension  (-0.013) | Obesity  (-0.013) | Dyslipidemia  (-0.012) | Diabetes (0.0053) | FH of MI/stroke (-0.00088) |
| Hematocrit | 0.299 | 0.247 | -0.0522 | -17.5 | Smoking  (-0.030) | Hypertension  (-0.012) | Diabetes  (-0.0052) | Dyslipidemia  (-0.0030) | Obesity  (-0.0014) | FH of MI/stroke (-0.00076) |

Model 1: adjusted for age

Model 2: additionally adjusted for traditional cardiovascular risk factors.

M stands for model, CVRF for cardiovascular risk factors, SI for stiffness index, BM for biomarker, CRP for C-reactive protein, WBCC for white blood cell count, IL-18 for interleukin-18, IL-1RA for interleukin-1 receptor antagonist, FH for family history, and MI for myocardial infarction.

**Supplemental Table 4. Influence of Cardiovascular Risk Factors on the Association Between Stiffness Index and Circulating Biomarkers in Women**

|  | **β-Estimate** | | **Difference in β-Estimate (M1 vs M2)** | | **Influence of CVRF on the association between SI and BM**  **(Absolute Difference in β-Estimate per Risk Factor, M1 vs M2)** | | | | | |
| --- | --- | --- | --- | --- | --- | --- | --- | --- | --- | --- |
|  | Model 1 (M1) | Model 2  (M2) | Absolute | Relative,% | 1  (Strongest) | 2 | 3 | 4 | 5 | 6  (Weakest) |
| CRP | 0.0902 | 0.0507 | 0.0395 | -43.8 | Hypertension  (-0.011) | Smoking  (-0.0081) | Obesity  ( -0.076) | Dyslipidemia  (-0.0023) | FH of MI/stroke (0.0003) | Diabetes (0.000011) |
| WBCC | 0.116 | 0.0555 | 0.0606 | -52.2 | Smoking  (-0.039) | Hypertension  (-0.012) | Obesity  (-0.0036) | Dyslipidemia  (-0.0022) | Diabetes  (0.00035) | FH of MI/stroke (0.000073) |
| Neopterin | 0.00471 | 0.00617 | 0.00146 | 31.0 | Smoking (0.0034) | Dyslipidemia  (-0.0012) | Diabetes  (-0.0010) | FH of MI/stroke (0.00093) | Hypertension  (0.00010) | Obesity (-0.000010) |
| IL-18 | 0.0137 | -0.0111 | 0.0248 | -181 | Smoking  (-0.0097) | Dyslipidemia  (-0.0043) | Hypertension  (-0.0041) | Diabetes (-0.0023) | Obesity  (-0.00061) | FH of MI/stroke (0.00035) |
| IL-1RA | 0.0916 | 0.0586 | 0.0330 | -36.0 | Hypertension  (-0.0093) | Dyslipidemia  (-0.0079) | Smoking  (-0.0050) | Obesity  (0.0036) | Diabetes (-0.0029) | FH of MI/stroke (0.00091) |
| Fibrinogen | 0.104 | 0.0607 | 0.0434 | -41.7 | Smoking  (-0.019) | Hypertension  (-0.0070) | Obesity  (-0.0064) | Dyslipidemia  (-0.0034) | FH of MI/stroke (-0.00021) | Diabetes (0.000021) |
| Hematocrit | 0.215 | 0.193 | 0.0219 | -10.2 | Smoking  (-0.016) | Hypertension  (-0.0042) | Obesity  (-0.0011) | Diabetes  (0.00072) | Dyslipidemia  (-0.00055) | FH of MI/stroke (-0.0000034) |

Model 1: adjusted for age

Model 2: additionally adjusted for traditional cardiovascular risk factors.

M stands for model, CVRF for cardiovascular risk factors, SI for stiffness index, BM for biomarker, CRP for C-reactive protein, WBCC for white blood cell count, IL-18 for interleukin-18, IL-1RA for interleukin-1 receptor antagonist, FH for family history, and MI for myocardial infarction.

|  | **Population-based sample with measurable SI (n=12,650)** | | | |
| --- | --- | --- | --- | --- |
|  | **Subsample without cardio-vascular risk factors/prevalent CVD (n=2,743)** | | **Subsample with cardio-**  **vascular risk factors/prevalent CVD (n=9,907)** | |
|  | Men | Women | Men | Women |
| n | 1,101 | 1,642 | 5,477 | 4,430 |
| Age, years | 49.4±10.5 | 48.7±9.9 | 55.5±10.9 | 55.5±10.8 |
| BMI, kg/m^2^ | 25.0±2.4 | 23.6±2.8 | 28.3±4.3 | 27.9±5.9 |
| Systolic BP, mmHg | 123±9 | 117±10 | 135.5±16.1 | 131.4±18.2 |
| Diastolic BP, mmHg | 78.8±5.9 | 76.3±6.7 | 85.0±9.6 | 82.6±9.6 |
| Heart rate, bpm | 65.4±9.3 | 69.3±9.4 | 68.4±11.1 | 70.1±10.5 |
| Hypertension, % | - | - | 63.3 | 56.7 |
| Diabetes mellitus, % | - | - | 10.4 | 7.0 |
| Smoking, % | - | - | 25.0 | 25.0 |
| Dyslipidemia, % | - | - | 43.9 | 29.0 |
| Obesity, % | - | - | 30.9 | 32.0 |
| FH of MI/stroke, % | - | - | 24.6 | 32.7 |
| History* of CAD, % | - | - | 7.0 | 2.5 |
| History* of MI, % | - | - | 4.8 | 1.7 |
| History* of CHF, % | - | - | 1.4 | 1.6 |
| History* of Stroke, % | - | - | 2.6 | 1.5 |
| History* of PAD, % | - | - | 3.8 | 4.1 |
| History* of CKD % | 0.3 | 0.7 | 1.3 | 1.0 |
| History* of COPD, % | 2.1 | 3.2 | 4.6 | 6.5 |
| OC intake, % | - | 10.4 | - | 5.2 |
| HRT, % | - | 6.2 | - | 8.5 |
| Menopause, % | - | 44.5 | - | 72 |
| ESC SCORE^†^, % | 1.0 (0.0/2.00) | 0.0 (0.0/1.0) | 2.0 (1.0/6.0) | 1.0 (0/3.00) |
| FRS^‡^, % | 6.1 (3.8/10.8) | 2.7 (1.7/4.6) | 18.2 (10.25/29.28) | 8.31 (4.38/14.44) |
| SI, m/s | 7.43±2.23 | 6.19±1.43 | 8.53±2.30 | 6.84±1.74 |
| CRP, mg/l | 0.8 (0.5/1.7) | 1.1 (0.5/2.1) | 1.60 (0.69/3.10) | 1.90 (0.89/3.90) |
| WBCC, 10^9^/l | 6.1 (5.2/7.3) | 6.6 (5.6/7.7) | 7.00 (5.87/8.30) | 7.10 (6.00/8.43) |
| Neopterin, pmol/l^#^ | 5.2 (4.6/6.1) | 5.1 (4.6/6.1) | 5.50 (4.70/6.48) | 5.40 (4.75/6.40) |
| IL-18, pg/ml^§^ | 223 (178/278) | 188 (156/237) | 248 (196/319) | 211 (171/271) |
| IL-1RA, pg/ml^**^ | 255 (191/327) | 288 (221/372) | 318.2 (244.7/425.4) | 346.0 (256.0/464.4) |
| Fibrinogen, mg/dl | 281 (248/324) | 299 (264/341) | 322 (279/376) | 336 (292/390) |
| Hematocrit, % | 43.3±2.8 | 39.8±2.7 | 43.8±2.9 | 40.4±2.7 |

**Supplemental Table 5. Demographic, Clinical and Laboratory Characteristics of Study Participants with Measurable Stiffness Index**

^*^ Medical records. ^†^ German version of the ESC SCORE. ^‡^ Framingham general CVD

Risk Score. Data are expressed as mean ± SD or medians (Q1/Q3).

^#^n=3,548 (1,886 men/1,662 women); ^§^n=4,078 (2,245 men/1,833 women); ^**^n=4,442 (2,337 men/2,105 women).

BMI stands for body mass index; BP for blood pressure; bpm for beats per minute; FH for family history; CAD for coronary artery disease; MI for myocardial infarction; CHF for congestive heart failure; PAD for peripheral artery disease; CKD for chronic kidney disease; COPD for chronic obstructive pulmonary disease; OC for oral contraceptives; HRT for hormone replacement therapy; FRS for Framingham risk score; SI for stiffness index; CRP for C-reactive protein; WBCC for white blood cells count; IL-18 for interleukin-18; IL-1RA for interleukin-1 receptor antagonist.

**Part C. SUPPLEMENTAL FIGURES**

**Supplemental Figure 1. Assessment of Arterial Stiffness by Digital Photoplethysmography**

**A) Calculation of Stiffness Index B) Assessment of “Very Stiff” Vasculature**

**
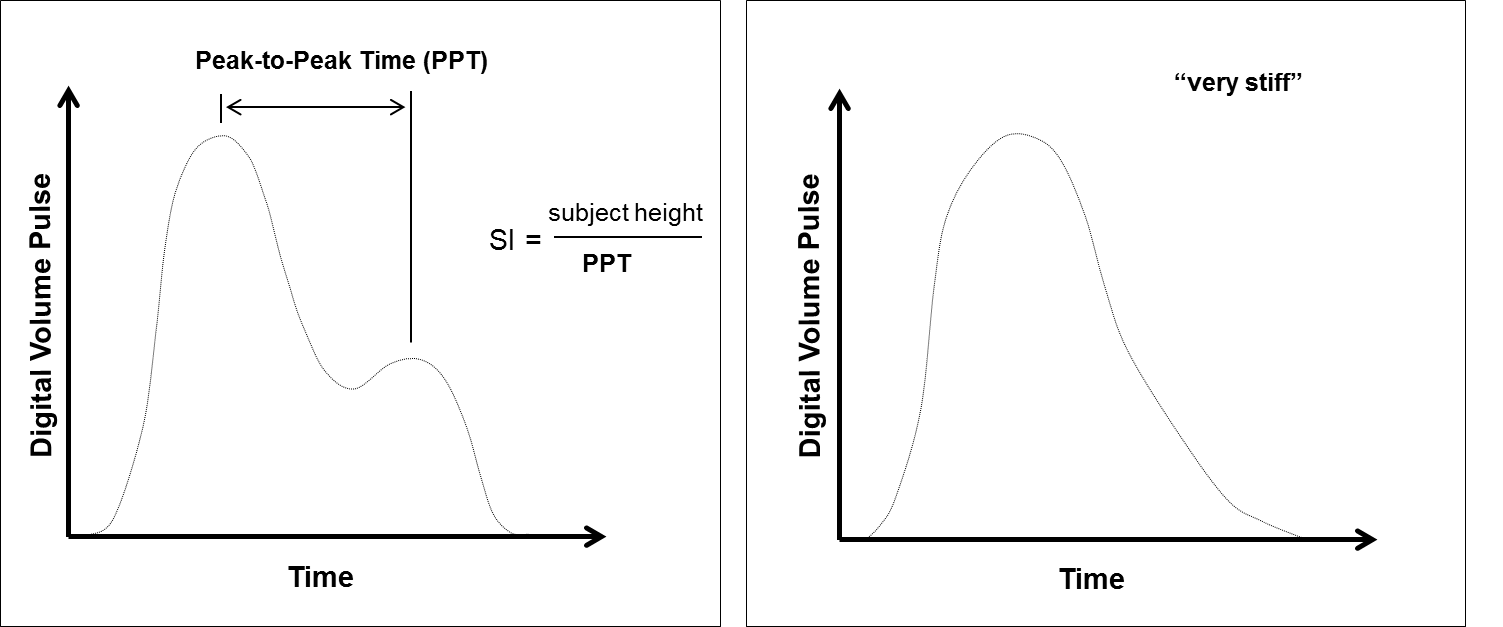
**

**Supplemental Figure 2. Ten-year Risk for CVD According to the Framingham CV Risk Score by Tertiles of Stiffness Index and (A) C-reactive Protein and (B) Fibrinogen**


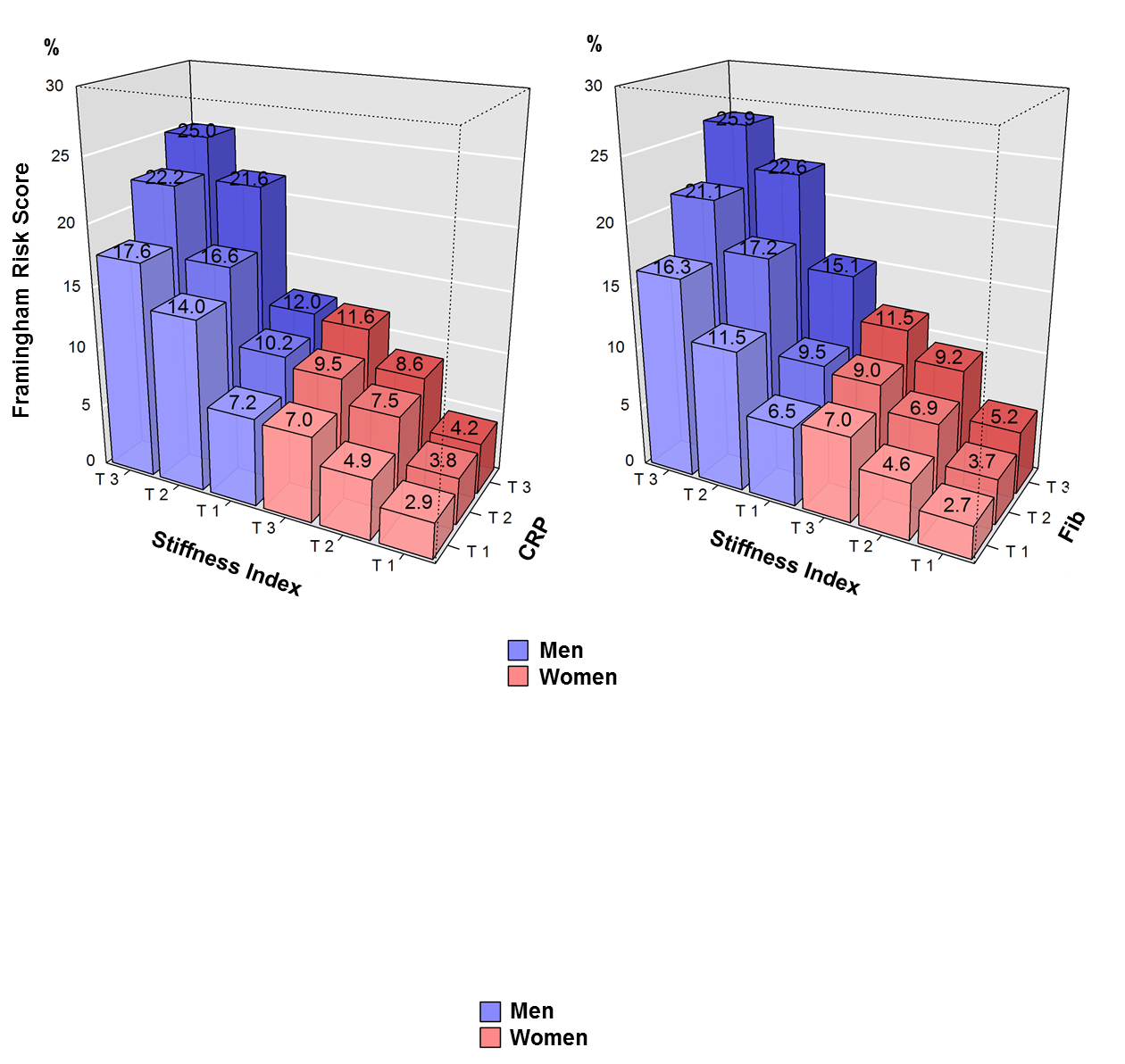


**A) B)**

### CRP stands for C-reactive protein; Fib for Fibrinogen.Supplemental Figure 3. Survival Over 8 Years According to Values Below or Equal and Above the Median for Stiffness Index and Circulating Biomarkers

###
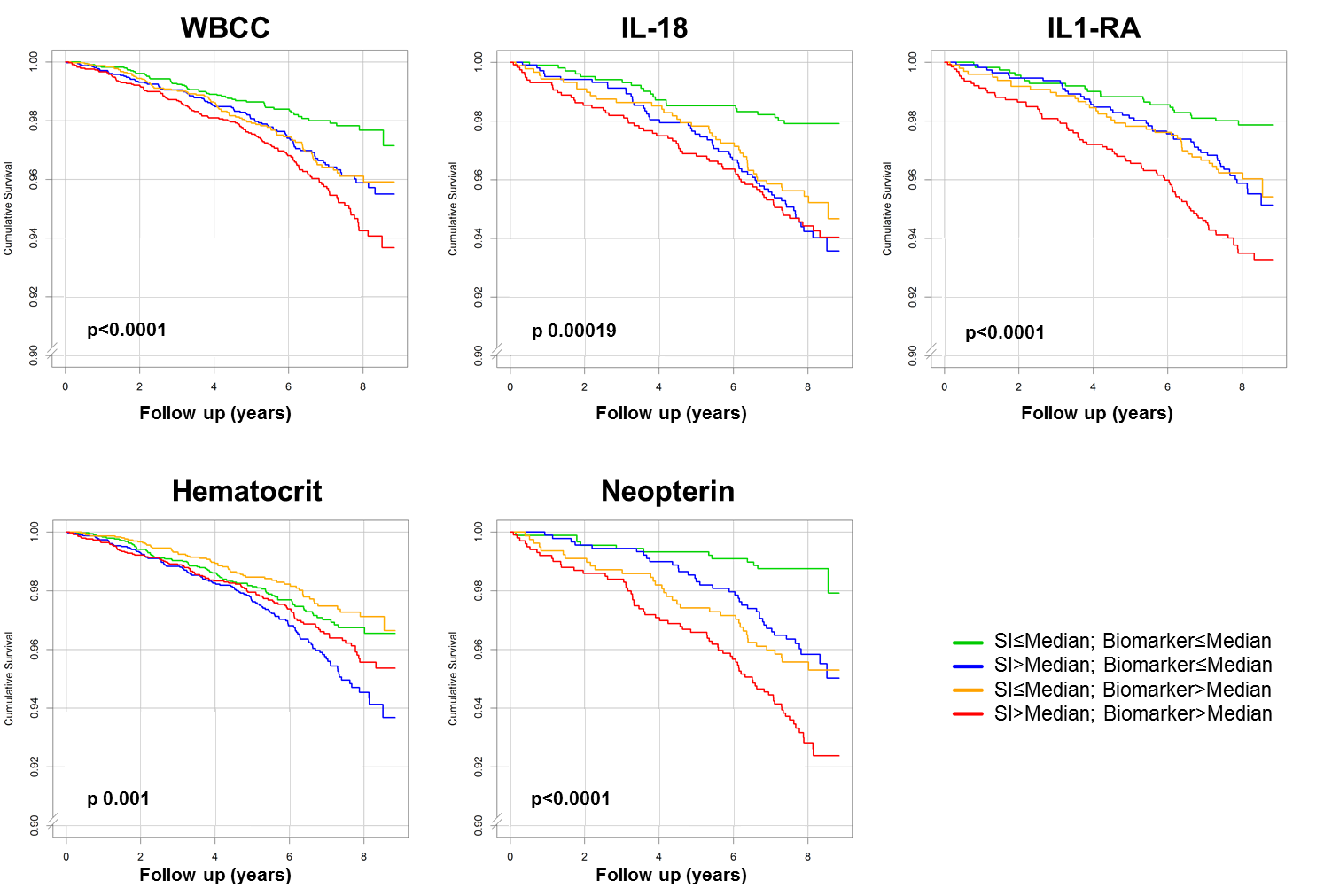


### The panels display Kaplan-Meier Curves for a 8-year follow-up period. SI stands for stiffness index, WBCC for white blood cells count, IL-18 for interleukin-18, and IL-1RA for interleukin-1 receptor antagonist.
